# Sex differences in outcomes of first episode psychosis: results from an early intervention service

**DOI:** 10.3389/fnbeh.2025.1642460

**Published:** 2025-09-08

**Authors:** Ilaria Domenicano, Alice Onofrio, Martina Citton, Ludovica Vecchioni, Domenico De Donatis, Raffaella Bertelli, Franca Emanuelli, Luigi Grassi, Maria Ferrara

**Affiliations:** ^1^Department of Neuroscience and Rehabilitation, Institute of Psychiatry, University of Ferrara, Ferrara, Italy; ^2^Department of Mental Health and Pathological Addiction, Local Health Trust (AUSL) Ferrara, Ferrara, Italy; ^3^Department of Psychiatry, Yale School of Medicine, New Haven, CT, United States

**Keywords:** early intervention, first-episode psychosis, psychotic disorders, women, sex differences

## Abstract

**Background:**

Early intervention services (EIS) for first-episode psychosis (FEP) play a key role in shaping a better disease trajectory for both affective and non-affective psychosis. Psychotic disorders tend to present sex differences both from an epidemiological and clinical perspective.

**Aims:**

The primary aim of this study is to investigate sex-based differences in outcome of patients admitted to EIS for FEP, analysing clinical differences and recovery rates during a 24 months long follow-up.

**Methods:**

A longitudinal cohort study was conducted. Patients were those admitted to the EIS in Ferrara between 2012 and February 27th, 2025 who met the following enrolment criteria: (a) diagnosed with affective or non-affective FEP; (b) not being treated for more than 24 months; (c) absence of intellectual disability; (d) aged between 18 and 35 years; (e) absence of organic psychosis. Socio-demographic and clinical characteristics were collected at program admission. The HoNOS (Health of the Nation Outcome Scale) was administered at baseline and every 6 months for the 24 months follow-up to compare sex differences in terms of symptoms severity and clinical recovery (HoNOS total score <8). Outcomes over time were compared between groups using mixed effects models repeated measures analysis of variance (MMRM).

**Results:**

A total of 174 patients were included in the study, most were males (74.1%), and most men vs. women were born in Italy (81.4% vs. 66.7%, *p* = 0.04). At admission, men had significantly higher rates of cannabis use (56.6% vs. 22.2%), tobacco use (62% vs. 28.9%), and alcohol misuse (51.2% vs. 15.5%) (*p* < 0.001). Men, compared to women, at 6 and 12 months showed significantly lower clinical severity than women (11.9 vs. 14.5, *p* = 0.03; 9.4 vs. 11.9, *p* = 0.05 respectively), and higher probability of being in recovery at 12 months (*p* = 0.04), indicating a faster clinical improvement. At 24-month, more men than women were NEET (Not in Education, Employment or Training) (26.3% vs. 8%, *p* = 0.04).

**Conclusion:**

Overall, our study highlighted significant sex differences both at admission as well as in outcomes. Men tend to improve more rapidly than women, then reaching a plateau with no substantial differences between sexes at 24 months. Further studies should identify sex-specific outcome predictors that could help in early patients’ identification, thus leading to improve clinical trajectories and long-term prognosis.

## 1 Introduction

First-episode psychosis (FEP) is mainly defined as the first appearance of positive psychotic symptoms, including hallucinations, delusions, and disorganized behavior (Breitborde et al., 2009). The median age for psychosis onset has frequently been reported to be 22–23 years (Kessler et al., 2007; Drake et al., 2016), which is a crucial time to self-define and shape one’s adult life. Because of this critical time interval, FEP hinders academic and career advancement, interferes with family and social networks, and negatively affects the subject’s social relations (Albin et al., 2021) and functioning (McGlashan, 1999).

While there is strong evidence on factors that influence FEP outcomes, such as duration of untreated psychosis (DUP) (McGlashan, 2000; Drake et al., 2000; Marshall et al., 2005; Penttilä et al., 2014; Dama et al., 2019a; Golay et al., 2023), admission to a specialized FEP program (Correll et al., 2018), cannabis use (Ricci et al., 2021; Kline et al., 2022), involvement of families in treatment (Doyle et al., 2014), and adherence to pharmacological treatment (Raghavan et al., 2019; Daneault et al., 2019), less is known about the influence of patients’ sex on prognosis. Although sex-driven exposure to risk factors, illness presentation and progression have been long studied in patients with chronic psychosis (Ochoa et al., 2012), strong evidence on how sex may affect FEP patients’ disease trajectory is lacking (Ferrara and Srihari, 2021). Most studies have in fact included mostly patients affected by chronic non-affective psychosis, such as schizophrenia, not to mention the majority involves retrospective data collection and chronic or mixed samples. Such studies have suggested better outcomes in women, since they displayed better premorbid functioning (Shtasel et al., 1992; Morgan et al., 2008; Brand et al., 2022), a more favorable global functioning during illness course (Grossman et al., 2008), lower disability rates (Usall et al., 2002), and a less frequent comorbid cannabis use (Ochoa et al., 2012).

Regarding FEP, what is known so far is that non-affective psychosis tends to present later in females’ life than in male counterparts (Ferrara et al., 2024a; Naughton et al., 2024), with an onset typically around 25–35 years of age and a second peak around menopause (Cotton et al., 2009; Ochoa et al., 2012; González-Rodríguez et al., 2020; Naughton et al., 2024). By contrast, some studies have shown an earlier onset of psychotic symptoms in women compared to men, even in adolescents. This has been hypothesised as due to women’s stronger illness insight and tendency to help-seeking actions (Køster et al., 2008; Magnabosco et al., 2024). Given that men are more likely to have an earlier onset, they suffer from dramatically important social implications. At psychosis onset men usually present with lower education levels, worse premorbid social and occupational functioning, more severe baseline negative symptoms, more alcohol or cannabis abuse problems, frequent hospital admissions and lower compliance to treatment (Cotton et al., 2009; Dama et al., 2019b; Ayesa-Arriola et al., 2020). On the other hand, it has been strongly suggested that women with psychotic disorders tend to be underdiagnosed due to their more common presentation with affective symptoms (Cotton et al., 2009; Irving et al., 2021), which can mislead mental health professionals to diagnose them with affective psychosis (Cotton et al., 2009) more often than male counterpart, excluding them from most FEP programs or studies (Ferrara and Srihari, 2021). In fact, being a female appears to be a significant predictor of receiving a diagnosis of affective rather than non-affective psychosis (Tseliou et al., 2017). Gender-specific biases of this kind may contribute to disparities in treatment that disadvantage women, by excluding them from coordinated specialty FEP care, or by delaying appropriate pharmacological treatment thus prolonging DUP and jeopardizing their prognosis (Ferrara et al., 2025): in fact, men have been found to be prescribed more antipsychotics, while female are more commonly prescribed mood-stabilizers and antidepressants (Ferrara et al., 2024b) and less frequently long acting injectable or clozapine (Ferrara et al., 2024a; Ferrara et al., 2025).

Since FEP patients present to care differently based on sex, it follows that outcomes might also differ between the sexes; however, up-to-date literature shows inconclusive results. Men with FEP are typically attributed lower remission rates (Addington and Addington, 2008), poorer clinical course and greater functional impairment at outcomes (Bertelsen et al., 2009; Morgan et al., 2014; Thorup et al., 2014; Drake et al., 2016). On the other hand, women with FEP display better global functioning at follow-up compared to men (Thorup et al., 2014), and it has been hypothesised that women’s higher insight and adherence to treatment (Ventura et al., 2023; Thorup et al., 2014) could explain the different outcomes in FEP. Even after receiving treatment, men present a comorbid drug abuse more frequently (Chang et al., 2011) and are at higher risk of being hospitalized (Thorup et al., 2014).

More recent studies and metanalyses considering FEP patients who had been treated by FES in various countries worldwide have shown no sex-related differences in symptoms at care presentation (Tseliou et al., 2017), quality of life (Hong et al., 2023), functional outcomes (Dama et al., 2019b), recovery (Jääskeläinen et al., 2013), and global functioning (Ayesa-Arriola et al., 2020).

Therefore, the aim of this study is to investigate sex-based differences in FEP admitted to an early intervention service, by looking at both clinical presentation as well as clinical outcomes inclusing global symptoms severity, and clinical recovery at 12 and 24 months after admission to the FEP program.

To our knowledge, this is the very first study in Italy which specifically targets sex-based differences in FEP outcomes.

## 2 Methods

### 2.1 Setting

The FEP care program in Ferrara, Northern Italy, is conceptualized as a specialty within generalist care model, and it draws its mission on the recommendations by the Regione Emilia-Romagna (2016) which were first embedded into clinical practice and then formalized locally in “PDTAs” (Percorso diagnostico terapeutico assistenziale—diagnostic and therapeutic care pathway) in 2019 (Belvederi Murri et al., 2021a; Ferrara et al., 2022). Such program provides clinical high risk (CHR) and FEP patients aged between 15 and 35 years with a specialized coordinated care treatment via pharmacological therapy, individual cognitive-behavioral therapy, physical health promotion and social inclusion programs, involving a psychiatrist and a case manager who provide care based on a stress-vulnerability explanation model for psychosis (Regione Emilia-Romagna, 2024). Referral to the program occurs via acute psychiatric hospital units, community mental health services (CMHC), ProMeco (psychologist in school), social services, and general practitioners (Ferrara et al., 2022).

### 2.2 Participants

This study included patients who were consecutively admitted to the FEP program in Ferrara from 2012 to February 17th, 2025, who were aged between 18–35 years, had been treated for psychosis for no longer than 24 months and had an ICD-9 diagnosis of affective (296.14, 296.24, 296.34, 296.44, 296.54, 296.64) or non-affective psychosis (295.0–295.95, 297.0–297.9, 298.0–298.9, 299.9). Patients who presented intellectual disability (IQ <50) or organic psychosis are not eligible for the program.

Although the FEP program in Ferrara includes both patients with FEP and CHR, this study only considered patients who were diagnosed with psychosis, therefore CHR individuals were excluded.

### 2.3 Measures

Demographic and clinical information were collected at the time of entry to the first-episode services (FES). Demographic information include age at psychosis onset, age at program admission, marital and parental status, country of birth, education level and employment status.

Recorded clinical information drawn from the Electronic Health Records included the psychosis diagnosis (affective/non-affective), referral to FES, having received treatment by Child and Adolescent Neuropsychiatry, admission to Pathological Addiction Service, family history of psychosis, cannabis use, alcohol use, tobacco use, psychopharmacological treatment, and DUP. DUP was calculated in months from the first evidence of at least one positive psychotic symptom to the beginning of an antipsychotic drug treatment for psychosis (Perkins et al., 2005). The type of psychopharmacological treatment was further classified as either oral administration or long-acting injectable (LAI) formulation. Information about family history of psychosis and cannabis, alcohol and tobacco use were retrieved from patient history, obtained through structured anamnesis conducted by trained clinicians and tox screen when available.

Follow-up assessments were conducted only for individuals who remained in care throughout the study period. Each patient’s overall symptomatology and functioning were evaluated by administering the Italian version of the Health of the Nation Outcome Scale (HoNOS) at baseline and every 6 months for 24 months. HoNOS is a hetero-administered scale (Preti et al., 2012) that includes 12 items, assessing both clinical and psychosocial features. Thus, in this study HoNOS has been used to assess both symptoms severity (the higher the score, the more severe the symptoms and overall clinical presentation) as well as the recovery status (by dichotomizing the total HoNOS score). The HoNOS is a hetero-administered scale consisting of 12 items, each one rated on a Likert-scale from 0 (no problem) to 4 (severe or very severe problem) (Preti et al., 2012). As a result, the higher is the score, the more severe are the symptoms and the overall clinical presentation. Patients were considered in clinical/functional recovery if total HoNOS score was <8 since this score corresponds to a level of symptomatology that allows the discharge of the patient from the CMHC with a referral to the general practitioner’s care (Prowse and Coombs, 2009). This scale has been recommended as the standard assessment by the Regional guidelines for FEP treatment (Regione Emilia-Romagna, 2016). The cut-off has been adopted by several FEP program in the Emilia-Romagna Region to determine the recovery status (Ferrara et al., 2019; Belvederi Murri et al., 2021b; Leuci et al., 2022; Ferrara et al., 2024c). Besides, given that items with no information available are assigned the score of 9, HoNOS evaluation has been considered invalid if at least one 9-scored item was present.

Being NEET (Not in Education, Employment or Training) at 12 and 24 months after FEP program admission was also used to assess patients’ outcomes (Srihari et al., 2015).

### 2.4 Statistical analysis

Bivariate analyses were performed to assess socio-demographic and clinical characteristics between the two groups (males vs. females) at admission to FES, while HoNOS scores and other clinical characteristics were analysed both at baseline and at 12 and 24 months follow up. The chi-squared test was conducted to analyse categorical data between sexes. The Mann–Whitney test was used to compare continuous non-normally distributed variables, while *t*-test was used to compare normally distributed data. The normality of continuous variables was detected through the Shapiro–Wilk test. For analysis using parametric statistics, DUP scores were log10 transformed because of positive data skewness.

Symptoms severity (average total HoNOS scores) and the recovery probability between the two groups at several endpoints (baseline, 6 months, 12 months, 18 months, and 24 months) were analyzed, 95% confidence intervals (CI) and effect size were reported. In summary, the two sex groups were then compared, at 12 and 24 months follow up evaluations, with respect to (a) proportion of patients who were considered to be in recovery status (HoNOS <8), (b) NEET status. Outcomes over time were compared between groups using mixed effects models repeated measures analysis of variance (MMRM). MMRM is advantageous because it includes all existing data in the model, without imputation or substitution of missing data. All existing data comprise the model.

Statistical analyses were conducted by using the software R. Statistically significant threshold was identified as *p*-value ≤0.05.

## 3 Results

### 3.1 Sample characteristics by sex at admission to the FEP program

**Figure 1:**
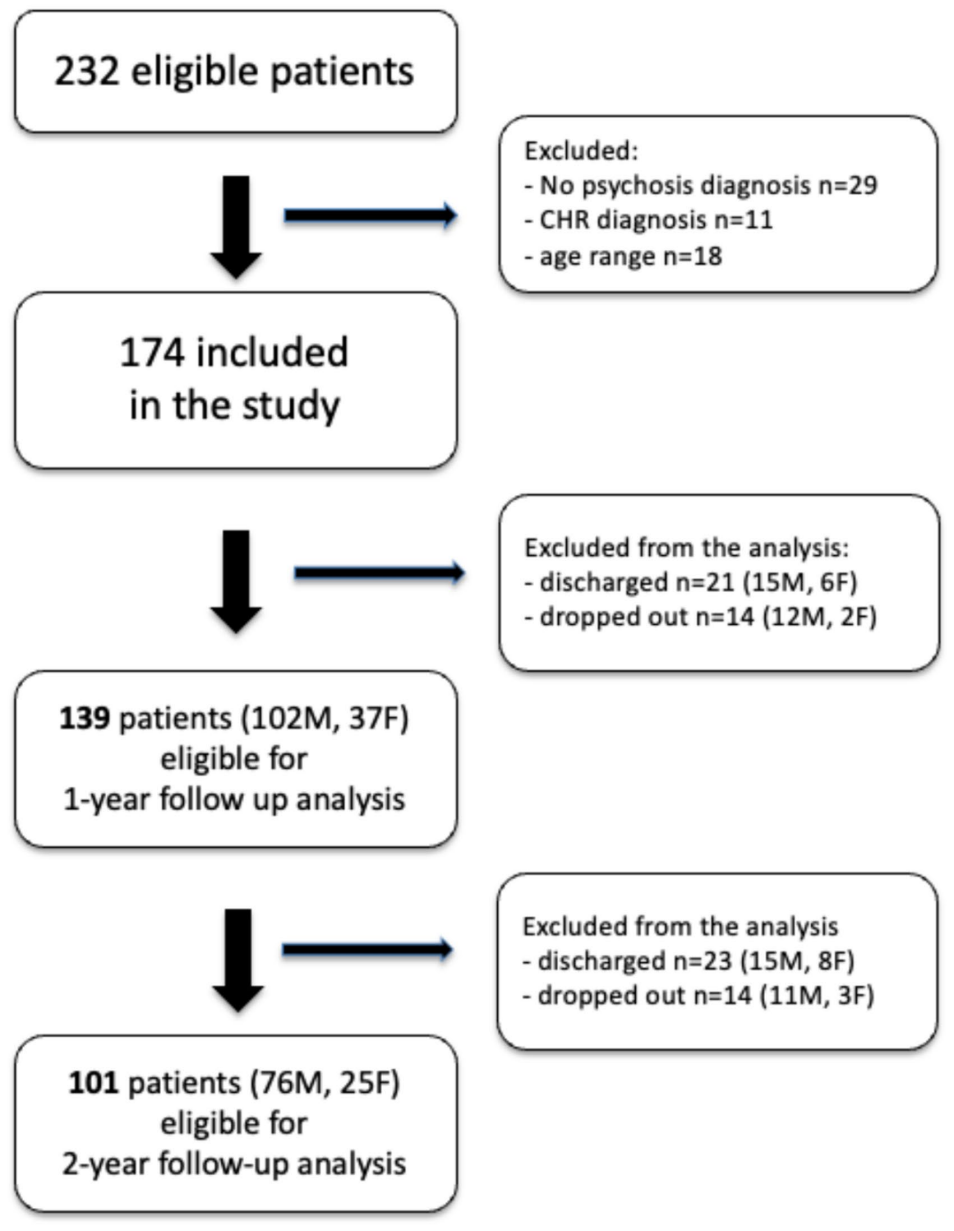
Sample selection flowchart.

During the observed time interval, a total of 219 patients were admitted to the FEP program in Ferrara, but only 174 met the study criteria and were eligible for the study. Figure 1 shows the study participants’ selection. Table 1 shows the demographic and clinical characteristics of the total sample at admission.

**Table 1:** Sex difference in demographic and clinical characteristics of the total sample at admission to the FEP program (*N* = 174).

	Total *N* = 174	Male *N* = 129 (74.1%)	Female *N* = 45 (25.9%)	*p*-value
Socio-demographic characteristics				
Age at program admission, years, mean (SD)	24.3 (4.3)	24.4 (4.2)	24.0 (4.5)	0.62^a^
Age at FEP onset, years, mean (SD)	22.9 (4.4)	23.0 (4.3)	22.7 (4.7)	0.79^a^
Born in Italy, yes, *N* (%)	135 (77.6)	105 (81.4)	30 (66.7)	0.04^b^
Marital status				0.18^b^
Single	168 (96.6)	126 (97.7)	42 (93.3)	
Married	3 (1.7)	1 (0.8)	2 (4.4)	
Cohabitant	1 (0.6)	1 (0.8)	0 (0.0)	
Divorced	1 (0.6)	0 (0.0)	1 (2.2)	
Other	1 (0.6)	1 (0.8)	0 (0.0)	
Has children, yes, *N* (%)	4 (2.3)	2 (1.5)	2 (4.4)	0.26^b^
Living situation, *N* (%)				0.15^b^
With parents	145 (83.3)	112 (86.8)	33 (73.3)	
Alone or with flatmates	21 (12.1)	12 (9.3)	9 (20.0)	
With partner and/or children	3 (1.7)	1 (0.8)	2 (4.4)	
Other	2 (1.1)	1 (0.8)	1 (2.2)	
Homeless	2 (1.1)	2 (1.5)	0 (0.0)	
Temporary accommodation provided by social services	1 (0.6)	1 (0.8)	0 (0.0)	
Education level, *N* (%)				0.35^b^
Short-cycle degree	17 (9.8)	11 (8.5)	6 (13.3)	
Master’s degree	6 (3.4)	3 (2.3)	3 (6.7)	
Lower secondary school	65 (37.4)	51 (39.5)	14 (31.1)	
Upper secondary school	83 (47.1)	61 (47.3)	21 (46.7)	
Unknown	4 (2.3)	3 (2.3)	1 (2.2)	
Employment status, *N* (%)				
Student (yes)	55 (31.6)	38 (29.5)	17 (37.8)	0.30^b^
Employed (yes)	63 (36.2)	49 (38.0)	14 (31.1)	0.44^b^
NEET (yes)	59 (33.9)	45 (34.9)	14 (31.1)	0.69^b^
Clinical characteristics				
DUP (weeks), mean (SD)	42.5 (90.4)	41.6 (93.4)	44.9 (82.2)	0.82^b^
logDUP (weeks), mean (SD)	2.5 (1.6)	2.4 (1.6)	2.7 (1.6)	0.25^a^
Non-affective psychosis diagnosis (yes), *N* (%)	146 (83.9)	108 (83.7)	38 (84.4)	0.91^b^
Referral to FEP, *N* (%)				0.30^b^
Relative	4 (2.3)	3 (2.3)	1 (2.2)	
GP	10 (5.7)	7 (5.4)	3 (6.7)	
Psychiatric ward	86 (49.4)	69 (53.5)	17 (37.8)	
Private specialist	2 (1.1)	2 (1.6)	0 (0.0)	
Community mental health centre	66 (37.9)	43 (33.3)	23 (51.1)	
Other	4 (2.3)	4 (3.1)	0 (0.0)	
Missing	2 (1.1)	1 (0.8)	1 (2.2)	
Antipsychotic treatment, *N* (%)				0.07^b^
None	9 (5.2)	6 (4.6)	3 (6.7)	
Only LAI	20 (11.5)	17 (13.2)	3 (6.7)	
Only oral	126 (72.4)	88 (68.2)	38 (84.4)	
Both	19 (10.9)	18 (13.9)	1 (2.2)	
Antipsychotic treatment, *N* (%)				0.01^b^
At least one LAI	39 (23.6)	35 (28.5)	4 (9.5)	
Only oral	126 (76.4)	88 (71.5)	38 (90.5)	
Cannabis use, yes, *N* (%)	83 (47.7)	73 (56.6)	10 (22.2)	<0.001^b^
Alcohol abuse, yes, *N* (%)	73 (41.9)	66 (51.2)	7 (15.5)	<0.001^b^
Family history of psychosis, yes, *N* (%)	59 (40.4)	44 (40.0)	15 (41.7)	0.86^b^
Received treatment by child and adolescent neuropsychiatry (yes), *N* (%)	24 (13.8)	14 (10.8)	10 (22.2)	0.06^b^
HoNOS total score (1–12), mean (SD)	18.3 (6.8)	18.0 (6.9)	18.9 (6.7)	0.49^a^

FEP, first-episode psychosis; NEET, not employed, education and training; DUP, duration of untreated psychosis (calculated as the time, in weeks, from the onset of the first psychotic symptom to the time suitable treatment is obtained); GP, general practitioner; LAI, long acting injectable antipsychotic; HoNOS, Health of the Nation Outcome Scale.

a Mann–Whitney test.

b Chi-squared test.

In the study sample most were of male sex (74.1%), and single (96.6%). Women, compared to men, were more often married (4.4% vs. 0.8%) and had children (4.4% vs. 1.5%). No substantial disparities were found in terms of age at admission, education level or employment status. Of note, fewer women were born in Italy compared to men (66.7% vs. 81.4%, *p* = 0.04).

Regarding clinical characteristics, mean DUP in the whole sample was 42.5 weeks and it was longer in women compared to men (44.9 vs. 41.6 weeks, *p* = 0.82), although not statistically significant. Most of the sample (83.9%) had been diagnosed with non-affective psychosis, but no differences were found between the two sexes in terms of psychosis type (affective vs. non-affective). Compared to men who were mostly referred by the inpatient unit (53%), women were mainly referred to the program by the community outpatient psychiatric service (51.1%). Women had previously received individual treatment by the Child and Adolescent Neuropsychiatry service more often than men (22.2% of women vs. 10.8% or men).

At admission to the program, considerably more men than women used cannabis (56.6% vs. 22.2%, *p* < 0.001), tobacco (62% vs. 28.9% *p* < 0.001) and had alcohol abuse problems (51.2% vs. 15.5%, *p* < 0.001). The average HoNOS total score at program entry was 18.3 (± 6.8) indicating a moderate overall illness severity, but no significant difference was observed between the sexes. The pharmacological treatment at admission to the program differed between the sexes: specifically, LAI antipsychotics were prescribed more frequently to men than to women (28.5% vs. 9.5%, respectively), whereas women were more often prescribed oral treatment only (90.5% in women vs. 71.5% in men).

### 3.2 Sex differences in outcomes at 12- and 24-months follow-up

A total of 139 (102 M, 79.1%) and 104 individuals (78 M, 76.5%) were eligible, respectively for the 12 and 24 month follow up (Figure 1): the attrition rate did not show any statistical difference between the sexes at both time points (*p* = 0.58, *p* = 0.52).

**Figure 2:**
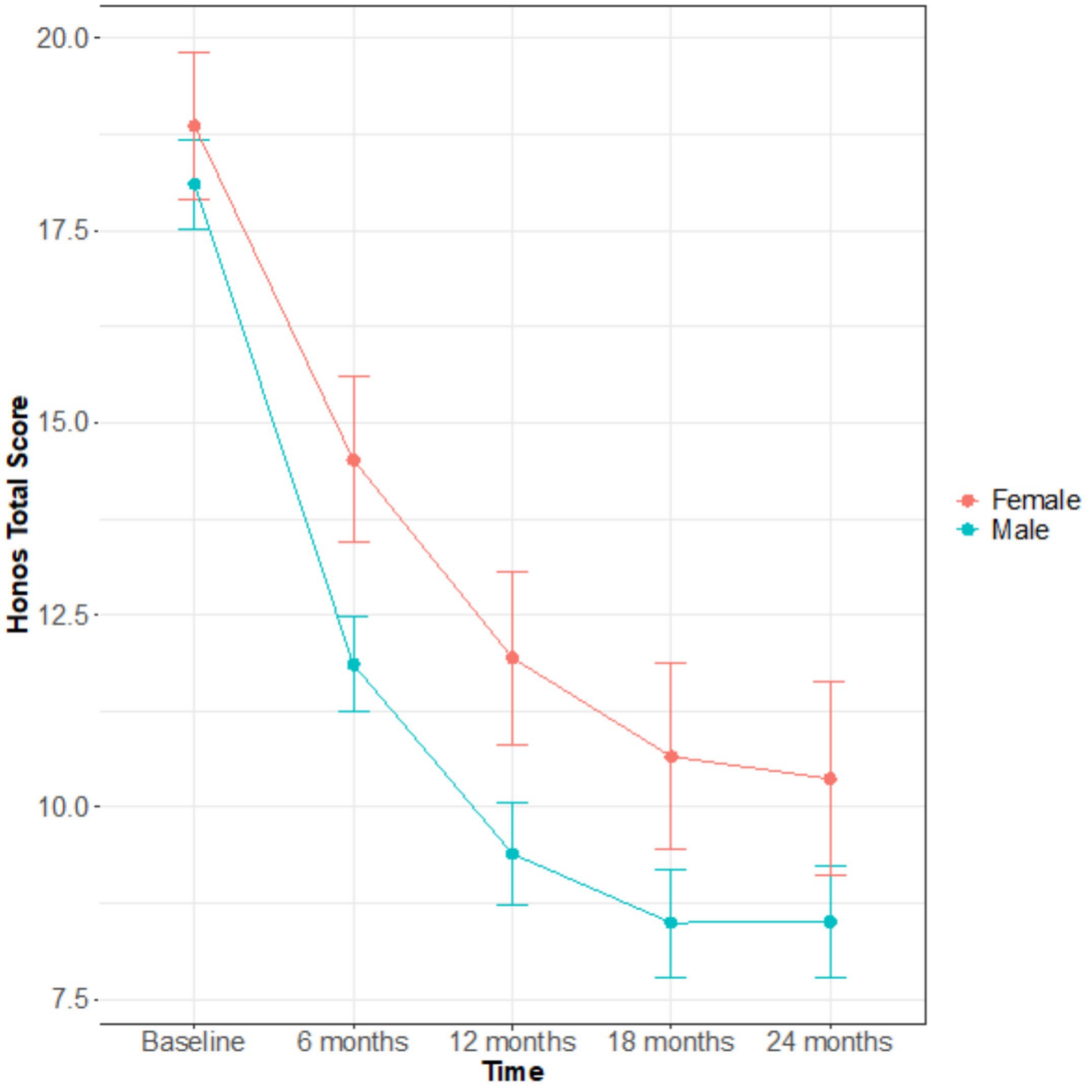
Sex difference in global symptom severity (HoNOS total score) over 24 months of follow up.

**Figure 3:**
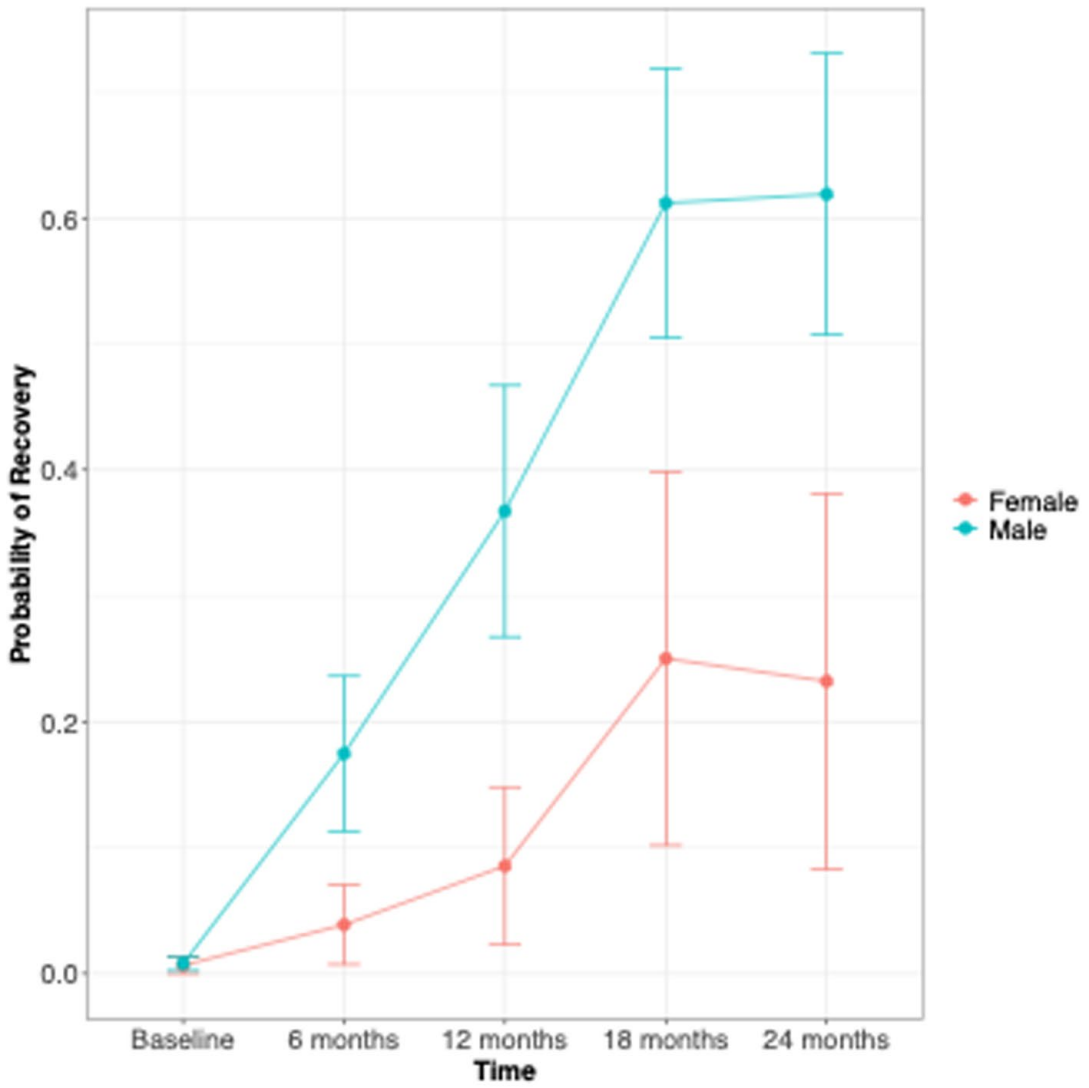
Sex difference in the proportion of patients who reached the Recovery status (HoNOS <8).

**Table 2:** Comparison of outcomes by sex at 12-months follow-up and 24-months follow-up.

	12 months follow up				24 months follow up			
	Total *N* = 139	Male *N* = 102 (73.4%)	Female *N* = 37 (26.6%)	*p*-value	Total *N* = 101	Male *N* = 76 (75.2%)	Female *N* = 25 (24.7%)	*p*-value
Employment status, *N* (%)								
Student (yes)	25 (18.0)	16 (15.6)	9 (24.3)	0.21^b^	16 (15.8)	10 (13.2)	6 (24.0)	0.10^b^
Employed (yes)	49 (35.3)	35 (34.3)	14 (37.8)	0.89^b^	47 (46.5)	33 (43.4)	14 (56.0)	0.17^b^
NEET (yes)	35 (25.2)	50 (24.5)	10 (27.0)	0.91^b^	22 (21.8)	20 (26.3)	2 (8.0)	**0.04** ^b^
Antipsychotic treatment, *N* (%)				0.96^b^				0.90^b^
Only oral	68 (48.9)	50 (49.0)	18 (48.6)		51 (50.5)	39 (51.3)	12 (48.0)	
Oral + LAI	17 (12.2)	12 (11.8)	5 (13.5)		9 (8.9)	6 (23.7)	3 (12.0)	
Only LAI	29 (20.9)	20 (19.6)	9 (24.3)		23 (22.8)	18 (23.7)	5 (20.0)	
None	12 (8.6)	9 (8.8)	3 (8.1)		14 (13.9)	11 (14.5)	3 (12.0)	
Antipsychotic treatment, *N* (%)				0.80^b^				0.89^b^
At least one LAI	46 (40.4)	32 (39.0)	14 (43.8)		32 (38.6)	24 (38.1)	8 (40.0)	
Only oral	68 (59.6)	50 (61.0)	18 (56.2)		51 (61.4)	39 (61.9)	12 (60.0)	
HoNOS total score, mean (SD)	9.7 (5.8)	9.2 (5.9)	11.3 (5.5)	0.09^a^	8.6 (5.6)	8.3 (5.7)	9.6 (5.3)	0.34^a^
Recovery (HoNOS <8), *N* (%)	41 (38.7)	34 (43.6)	7 (25.0)	0.08^b^	42 (52.5)	34 (56.7)	8 (40.0)	0.30^b^

NEET, not employed, education and training; LAI, long acting injectable antipsychotic; HoNOS, Health of the Nation Outcome Scale. Bold are the statistically significant results.

^a^Mann–Whitney test.

^b^Chi-squared test.

At both 12- and 24-months follow-up, the clinical outcomes investigated (clinical severity Figure 2, recovery status Figure 3) showed no significant differences between the sexes (Table 2). At both time points, men had a tendency to a lower overall illness severity [HoNOS total score 9.2 vs. 11.3, effect size = 0.37 (CI –0.07; 0.8); 8.3 vs. 9.6, effect size = 0.24 (CI –0.26; 0.75)], even though this difference was not significant. Of note, the only difference observed in terms of outcomes was a worst functional outcome observed in men vs. women at the 24 months time point: more men than women in fact were NEET at the 24 months follow-up (26.3% vs. 8%, *p* = 0.04).

Further analyses were performed to assess how the overall symptomatology (HoNOS total score) and recovery status (HoNOS total <8) progressed over time by sex (Table 3). At 6- and 12-months men had a significant lower HoNOS scores compared to women, respectively 11.9 ± 1.3 in males and 14.5 ± 2.1 in females at 6-months (*p* = 0.03), and 9.4 ± 1.3 in males and 11.9 ± 2.2 in females at 12-months (*p* = 0.05), so men seem to improve faster than women. Even though men scored lower than women at all endpoints, baseline, 18 and 24 months were not statistically significant.

**Table 3:** Multi model for repeated measure (MMRM) derived (group-sex-by time interactions) for the outcome measure for the sex groups.

Outcome	Male	Female	*p* ^a^	*p* ^b^
Baseline				
HoNOS tot, 1–12, mean (CI)	18.1 (17.0, 19.3)	18.9 (17.0, 20.8)	0.50	
Recovery, probability (CI)	0.008 (0.002, 0.03)	0.006 (0.001, 0.05)	0.87	
6-month				
HoNOS tot, 1–12, mean (CI)	11.9 (10.6, 13.1)	14.5 (12.4, 16.6)	**0.03**	
Recovery, probability (CI)	0.2 (0.1, 0.3)	0.04 (0.0, 0.2)	0.06	
12-month				
HoNOS tot, 1–12, mean (CI)	9.4 (8.1, 10.7)	11.9 (9.7, 14.2)	**0.05**	
Recovery, probability (CI)	0.37 (0.20, 0.6)	0.08 (0.02, 0.3)	**0.04**	
18-month				
HoNOS tot, 1–12, mean (CI)	8.5 (7.1, 9.9)	10.7 (8.3, 13.0)	0.12	
Recovery, probability (CI)	0.6 (0.4, 0.8)	0.2 (0.1, 0.6)	0.09	
24-month				
HoNOS tot, 1–12, mean (CI)	8.5 (7.1, 9.9)	10.4 (7.9, 12.8)	0.20	0.52
Recovery, probability (CI)	0.62 (0.4, 0.8)	0.23 (0.06, 0.6)	0.08	0.40

a Bonferroni adjustment for multiple comparisons. Bold are the statistically significant results.

b *p*-values for the group (male vs. female) by time interaction effect over the 24 months of treatment.

**Figure 4:**
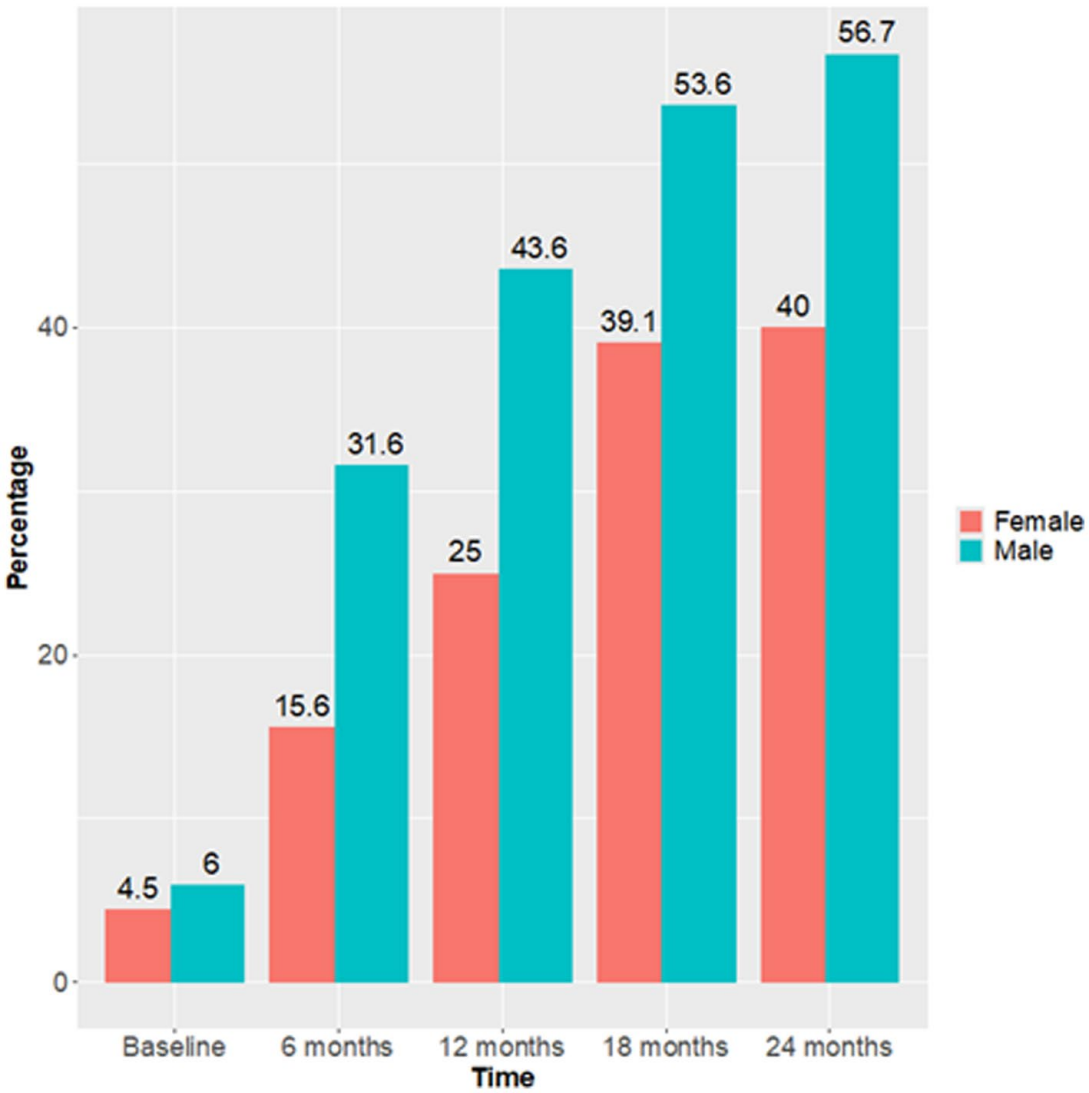
Sex difference in the proportion of patients who reached the Recovery status (HoNOS <8) over 24 months follow up.

Also, only at the 12-month follow up men had a statistically significant higher recovery probability than women [*p* = 0.04, effect size = 0.16 (CI 0.01; 0.33)]. Overall, the probability of recovery increased more rapidly in men than in women at the 6- and 12-month follow-ups, but the trend became similar between sexes at 18 and 24 months [effect size at 24 months = 0.14 (0.01; 0.3)] (Figure 3). The proportion of men who reached recovery status was greater than that of women at all endpoints (Figure 4).

However, the sex by time interaction effect over the 24 months of treatment was not significant for both illness severity (*p* = 0.52) as well as probability of being in recovery (*p* = 0.40).

## 4 Discussion

This study aimed to examine sex-based differences in FEP patients’ presentation and outcomes after admission to a specialized FEP program at 12 and 24 months follow up evaluations.

At admission, significant sex-differences were mostly found in cannabis use, tobacco use and alcohol abuse (more common in men), results that are in line with most of the current literature (Ochoa et al., 2012; Ayesa-Arriola et al., 2020). Age at FEP onset and age at presentation to care did not differ between the sexes, as opposed with the results of other studies about age of onset in psychotic disorders (Eranti et al., 2013; Jongsma et al., 2019; Ferrara et al., 2024a). While these studies did not exclude subjects based on age, our findings could be biased at origin as the Program poses a limitation to enrolment age (i.e., upper limit of 35 years), thus excluding mostly women who usually experience their first episode later in life (Ochoa et al., 2012; Riecher-Rössler et al., 2018), as already reported in our previous study (Ferrara et al., 2024b). Of note, earlier onset in men has previously been linked to worst functioning at care presentation (i.e., lower education level) (Dama et al., 2019b): our findings corroborate this hypothesis, since women in our sample were often studying and had a higher educational level.

Although not significant, the mean DUP had a tendency of being longer in women compared to men (44.9 vs. 41.6 weeks), which is consistent with the previous findings that reported a longer DUP in women (Køster et al., 2008; Cascio et al., 2012; Melle et al., 2004; Thomas and Nandhra, 2009). Notwithstanding, many past literature findings have pointed out that men tend to present a longer DUP (Cascio et al., 2012; Ferrari et al., 2018). We hypothesised this result could be linked to men being keener on alcohol and cannabis use which would make it more plausible for them to be referred to psychiatric services after an acute drug-triggered psychotic episode characterized by aggressive or disruptive behavior (Ferrara and Srihari, 2021). On the other hand, the longer DUP in women in our sample could be explained by their less disorganized and aggressive behavior (Køster et al., 2008), not to mention their more frequent presentation with affective symptoms at program admission (Ferrara and Srihari, 2021; Cotton et al., 2009; Irving et al., 2021) which could lead to misdiagnosis and delay in admission to FES (Ferrara and Srihari, 2021).

The global symptom severity was significantly lower in men than women at 6- and 12-months endpoints, and recovery rates were higher in males than females at the 12 months endpoint. Such findings were unexpected given previous evidence supporting poorer clinical course (Grossman et al., 2006; Bertelsen et al., 2009; Morgan et al., 2014; Pang et al., 2016) and worst recovery rates in men (Novick et al., 2016). However, at 18 and 24 months the trend became similar between sexes at 18 and 24 months, suggesting that men get especially better during the first year after their psychotic onset then reaching a sort of plateau.

This findings could also be explained by the notion that it is possible that, even after initial improvement, men tended to have more persistent negative symptoms at follow up than women, as previously reported (Thorup et al., 2014). On the other hand, recovery in women seems to take more time and so does reduced symptoms severity. The reason behind women’s slowest recovery might be linked to differences in pharmacological choices, for example the significant less frequent use of LAIs in women in our sample at entrance (28.5% vs. 9.5%) that could have hindered the speed of their recovery. This may reflect a prescribing bias, whereby clinicians are more likely to offer LAIs to patients perceived as less adherent—often younger males—based on assumptions about greater medication adherence among women, even though current evidence does not support that notion (Santos-Casado and García-Avello, 2019; Zhou et al., 2016). On the other hand, the more frequent use of LAIs among male patients in our sample may help explain their faster initial symptom reduction and higher probability of recovery (Cervone et al., 2015). These hypotheses are supported by the observation that the proportion of women on LAI increases over time, and the difference in LAI vs. oral between the sexes is no longer significative in the 12 and 24 time points. These hypotheses must be tested by a mediation analysis with a larger sample size. LAIs are associated with improved treatment adherence, reduced relapse risk, and fewer hospitalizations compared to oral formulations (Kishimoto et al., 2021). LAIs bypass first-pass metabolism, resulting in greater bioavailability, and they also maintain more stable plasma concentrations, reducing peak-related side effects and potentially minimizing the need for frequent treatment adjustments (Vita et al., 2024).

Recovery rates did not differ between the sexes: both sexes showed a significant improvement in global symptoms severity (the average total HoNOS scored halved from baseline to 1 and 2 years follow up), and 52.5% of the total sample (56.7% M, 40% F) was considered as being in recovery after 2 years. However, it must be acknowledged that, being this an observational real world study, it is possible that the sample was underpowered to detect significant differences in outcomes between the sexes, thus this might affect the interpretation of negative findings.

Lastly, despite not showing significant differences in employment or education at admission to the program, women were more often employed than men at 24-months follow-up, which is consistent with many studies reporting higher global functioning rates in women compared to men (Thorup et al., 2014; Drake et al., 2016).

### 4.1 Strengths

Our study has several strengths. First, to our knowledge this is the first study to analyse sex differences in outcomes in FEP patients in Italy thus pioneering the research on how sex and gender could influence clinical trajectories in psychosis. Second, clinical and socio-demographic characteristics were examined in real-life clinical practice, offering a realistic depiction of clinical outcomes. The analysis highlighted some differences both at clinical admission as well as in outcomes that could help tailoring the extant FEP program to sex characteristics and needs. Men could benefit from gender-sensitive approaches that address stigma and acknowledge male-specific help-seeking behaviors, like improving engagement and retention in care in Pathological Addiction Services or facilitating access to vocational rehabilitation programs. On the other hand, women could benefit from more intensive interventions in the first 12 months after a first-episode psychosis, and quality improvement initiatives that take in consideration the potential gender biases in pharmacological choices, expecially regarding the prescription of LAI.

### 4.2 Limitations

Some limitations need to be taken into account when interpreting our results. First, individuals who discontinued care—either due to significant clinical improvement or for other reasons such as relocation or disengagement—were not included in the follow-up. Considering that a longer follow-up period is important when considering recovery as an outcome, this may be responsible for many non-statistically significant findings (Ferrara et al., 2024c). Second, in our sample, sex prevalence was uneven, with 74.1% men and 25.9% female, a proportion within this age range (18–35) that was expected (Ferrara et al., 2019; Belvederi Murri et al., 2023; Jongsma et al., 2018) but the attrition rate was similar for both the sexes. Moreover, the underrepresentation of women in our sample may also be due to their scarce presence in FEP programs due to the restricted age criteria (Ferrara and Srihari, 2021; Ferrara et al., 2024a; Ferrara et al., 2024b), which excludes women who have their onset at a more mature age (Ferrara et al., 2024a; Ferrara et al., 2024b; Salvadé et al., 2024), thus leading to broaden sex iniquity (Lappin et al., 2016; Ferrara and Srihari, 2021; Seeman, 2021). This selection bias could limit the generalizability of the results to Services such as those in the UK which admit FEP above the 35 years old thereshold; however these findings apply to most FEP programs both at a national as well as international contest that admit only young adults up to age 35. Third, due to this study being observational, no strict cause-effect relation could be established, thus permitting to reveal only mere association between the variables. Moreover, the study design could explain the presence of missing data on older charts that could have influenced the results. Fourth, individuals were selected if their DUP was within 2 years, according to the regional recommendations, but differently from other international program; this might limit the generalizability of the results. Last, given that many patients were born in Italy and only around 20% are foreigner, results may not be generalizable to ethnically diverse samples that could be found in other geographic areas.

### 4.3 Clinical implication and future directions

To our knowledge this is the first study that showed a gender difference in timing of recovery trends, and this finding could help tailoring the pace and the quality of interventions to males and females needs. Poor vocational outcomes (NEET rates) were significantly higher in males than females, suggesting that symptomatic recovery does not necessarily equate to functional recovery, particularly in male patients (Ferrara et al., 2024c) as others have reported (Chang et al., 2011; Thorup et al., 2014). This highlights the need for psychosocial and vocational interventions tailored to support vocational attainment in men. This suggests that FEP programs should personalize identification strategies and treatment based on sex in order to detect high risk patients before or right after their psychosis onset, since this could lead to DUP reduction and improvement of life quality and global functioning, shaping a better disease trajectory. As a recent qualitative investigation has outlined, mental health providers are aware of possible sex differences in the epidemiology and presentation of FEP, however they also acknowledge the lack of specific training including the influence of the menstrual cycle on symptoms relapse and response to pharmacological treatment (McGinty et al., 2025).

Last, this study focused only on biological sex and did not consider gender and gender roles. Given that gender might differ from biological sex, it should be carefully assessed at admission as it could influence pathways to care, clinical presentation and outcomes (Ferrari et al., 2018).

## 5 Conclusion

In conclusion, our study found statistically significant sex-based differences in clinical presentation and clinical outcomes at 12 months but not at 24 months follow-up. FEP may benefit from different treatment approaches based on sex: while women might require closer monitoring for clinical improvement in the early phases, men may need targeted interventions to address the comorbid substance abuse and to support long-term functional recovery. Larger longitudinal studies are needed to better assess sex and gender disparities in clinical and functional outcomes among FEP patients.

## Data Availability

The datasets presented in this article are not readily available because privacy restrictions and local regulation. Requests to access the datasets should be directed to the corresponding author.
